# The Mediating Role of Mindful Parenting in the Relationship Between Parental Anxiety and Youth’s Emotional and Behavioral Difficulties

**DOI:** 10.1007/s10964-023-01752-3

**Published:** 2023-02-22

**Authors:** Maite Larrucea-Iruretagoyena, Izaskun Orue

**Affiliations:** Faculty of Health Sciences, University of Deusto, Bilbao, Spain

**Keywords:** Mindful parenting, Parental anxiety, Emotional and behavioral difficulties, Youth

## Abstract

One of the central questions in the theory of the intergenerational transmission of psychological symptoms is to identify whether parenting practices explain the transmission of psychological symptoms from parents to youth. This study examined the mediating mechanism of mindful parenting in the relationship between parental anxiety and youth’s emotional and behavioral difficulties. In three waves separated by six months, longitudinal data were collected from 692 Spanish youth (54% girls) aged between 9 and 15 years (*M*_age_ = 12.84 years, SD = 1.22 years at Wave 1) and their parents. Path analysis showed that maternal mindful parenting mediated the relationship between maternal anxiety and the youth’s emotional and behavioral difficulties. No mediating effect was found concerning fathers; however, marginal bidirectional relationships were obtained between paternal mindful parenting and youth’s emotional and behavioral difficulties. This study addresses one of the main concerns about the theory of intergenerational transmission using a multi-informant and longitudinal study design, concluding that maternal anxiety predicts less mindful parenting practices and these in turn predict youth’s emotional and behavioral difficulties.

## Introduction

The risk of transmitting psychological symptoms from parents to offspring has been widely studied. However, the mechanisms behind the intergenerational transmission of psychological symptoms remain largely unknown (Cerniglia & Cimino, 2020). Negative parenting practices and low positive parenting practices maintain the transmission of psychological symptoms from parents to offspring, which may affect the development of mental disorders in adolescence (Raballo et al., 2021). The field of study that can provide relevant information on how to break the transmission of psychological symptoms from parents to youth through positive parenting practices, such as mindful parenting, remains largely unexplored (Medeiros et al., 2016). This study aimed to answer one of the central questions of the theory of intergenerational transmission about the mechanism of mindful parenting in the transfer of psychological symptoms from parents to youth.

Almost all psychological theories agree with the importance of physical, social, and family environments for youth development (Marceau et al., 2022). Despite this recognition, this phenomenon has only been accurately explained by a few conceptual elaborations (Cerniglia & Cimino, 2020). One of the most recognized in this sense is the theory of the international transmission of psychological symptoms, which explains the risk of transmitting psychological symptoms across generations (Goodman, 2020). A multitude of studies have focused on demonstrating this theory, concluding that youth with parents with psychological symptoms have a higher risk of developing psychological symptoms than those with parents without any reported psychological symptoms (Gamliel et al., 2018; Schulz et al., 2021; Vismara et al., 2022). One of the variables that best predicts the development of psychological symptoms in youth is the psychological symptoms reported by their parents (Goodman et al., 2011).

The study of intergenerational transmission has mainly focused on the transmission of depression, trauma, or violence. In recent years, studies have highlighted the importance of analyzing the effects of other psychological disorders, such as anxiety (Goodman, 2020). Parental anxiety is a predictor of a youth’s internalizing symptoms (Spry et al., 2020). A meta-analysis has concluded that parental anxiety is a risk factor for youth in developing internalizing symptoms related to anxiety and depression disorders (Möller et al., 2016). One of the few longitudinal studies that analyzed this predictive path between parental anxiety and youth-internalizing symptoms reached the same conclusion (Johnco et al., 2021). Further research on paternal anxiety is necessary, as the vast majority of the samples included a higher percentage of mothers than fathers (Spry et al., 2020).

As the vast majority of studies have been conducted with mothers, maternal anxiety symptoms seem to affect a youth’s emotional and behavioral difficulties more than paternal anxiety symptoms. Given that mothers are the principal day-to-day caregivers, youth are more exposed to maternal anxiety symptoms than paternal ones (Ahmadzadeh et al., 2019). Even though several studies regarding this phenomenon have focused on the first stages of life, several authors have emphasized the importance of evaluating the effects of parental psychological symptoms on emotional and behavioral difficulties in youth, since the effect of parental psychological symptoms intensifies with offspring’s age because they are exposed longer to parental psychological symptoms (Möller et al., 2016).

Important changes occur in the parent–youth relationship during adolescence (Van der Bruggen et al., 2008); thus, other mechanisms that may explain the transmission of parental psychological symptoms to youth’s psychological symptoms should be understood. In recent years, some studies have analyzed the mediating role of certain mechanisms that can explain/maintain this predictive relationship. Parenting practices have been given special attention (Belsky et al., 2009). Some authors (Kerr & Capaldi, 2019; Raballo et al., 2021) indicate that the use of negative parenting practices (e.g., harassment, excessive control, and rejection) and low positive parenting practices (e.g., warmth, listening with attention, or positive reinforcement) maintains the relationship between parental psychological symptoms and youth’s psychological symptoms (Vafaeenejad et al., 2019). When parents are struggling with psychological symptoms, the quality of their parenting practices may decrease and, consequently, the youth may develop emotional and behavioral difficulties (Cerniglia & Cimino, 2020). Thus, parenting practices may mediate the relationship between parental anxiety and youth’s emotional and behavioral difficulties.

Even though the mediating mechanism of parenting practices is largely unknown, research has suggested that anxious parents tend to use more negative parenting practices and less positive parenting practices (Vafaeenejad et al., 2019). Some studies have concluded that negative parenting practices are related to a youth’s emotional and behavioral difficulties (Camisasca et al., 2022) and that positive parenting practices lead to better well-being outcomes (Chen et al., 2019).

Among these parenting practices, mindful parenting has sparked huge interest in the past decade. *Mindful parenting* refers to the parental ability to attend to an offspring and parent intentionally and non-judgmentally while being conscious about the present moment (Kabat-Zinn & Kabat-Zinn, 1997). Based on this concept, Duncan et al., (2009) proposed a model of mindful parenting and stressed how it promotes a high-quality parent‐offspring relationship because mindful parenting abilities allow parents to raise an offspring while considering both their and their offspring’s emotions, being sensitive to them and giving their full attention, and developing a high capacity of non-judgmental acceptance and compassion for their offspring and themselves (Potharst et al., 2017).

In recent years, studies have suggested that mindful parenting is related to an improved parent–youth relationship and well-being in both parents and youth (Parent et al., 2016). Mindful parenting practices benefit a youth’s emotional and behavioral problems; these practices are vulnerable to disruption due to the youth’s emotional and behavioral problems, suggesting a bidirectional relationship between these two parental and youth variables (Kim & Gonzales, (2021)). In addition, parents with higher anxiety symptoms showed lower levels of mindful parenting (Bögels et al., 2014). Mindful parenting was cross-sectionally related to youth’s secure perceptions of their relationship with their parents (Moreira et al., 2018) and low externalizing and internalizing behaviors among youth (Yang et al., 2022). For this reason, mindful parenting can be a mediator in the relationship between parental anxiety symptoms and youth’s emotional and behavioral difficulties. Studies that longitudinally analyzed this mediation with a multi-informant response design (mother, father, and youth) have not been conducted. One study has evaluated the mediation of mindful parenting between parental anxiety and offspring’s emotional and behavioral difficulties and found that maternal mindfulness mediates this relationship (Henrichs et al., 2021). This study was conducted with the mothers of toddlers, and only data collected from one informant were used. Several studies have indicated that longitudinal studies with multi-informant samples and a similar percentage of participating mothers and fathers are necessary (Johnco et al., 2021).

## Current Study

**Fig. 1:**
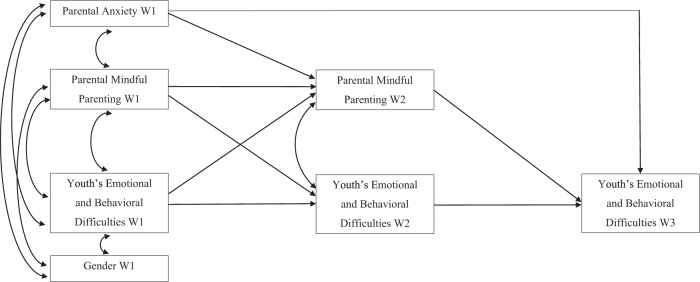
Conceptual model. W1 = Wave 1; W2 = Wave 2; W3 = Wave 3

Parental anxiety symptoms seem to be one of the most important predictors of the development of youth’s emotional and behavioral difficulties. Nonetheless, little is known about how mindful parenting practices may maintain this intergenerational transmission of psychological symptoms from parents to youth remains scarcely known. The main objective of the present study was to evaluate the mediating role of mindful parenting in the relationship between parental anxiety and youth’s emotional and behavioral difficulties through a multi-informant three-wave longitudinal design (Fig. 1). Hypothetically, low levels of mindful parenting may mediate the relationship between parental anxiety symptoms and youth’s emotional and behavioral difficulties. Two mediating hypothesized models were evaluated in the study, one with mother–youth dyads and the other with father–youth dyads.

## Methods

### Participants

A total of 692 youth (54% girls) and both parents of each youth were invited to participate in this longitudinal study composed of three waves: Wave 1 (W1), Wave 2 (W2), and Wave 3 (W3), which were conducted in 2021, May, 2021, November, and 2022, May, respectively. As parental participation was much lower compared with that of the youth (see Table 1), only the mother–youth dyads and father–youth dyads that accomplished the three-wave measures were included in this study. A total of 221 cases in which all three family members (mother, father, and youth) responded to the questionnaires in all three waves, 69 cases of mothers and youth responded in all waves, and 20 cases of fathers and youth participated in all three waves. Therefore, 290 mother–youth dyads and 241 father–youth dyads were included in the study. Youth at W1 were aged between 9 and 14 years (*M*_age_ = 12.84 years, *SD* = 1.22 years), mothers were between 31 and 63 years old (*M*_age_ = 46.72 years, *SD* = 4.6 years), and fathers were between 32 and 78 years old (*M*_age_ = 48.91 years, *SD* = 5.44 years). Regarding socioeconomic level, 23.45%, 19.05%, 16.77%, 13.12%, and 27.61% of the participants belonged to the low, medium-low, medium, medium-high, and high socioeconomic levels, respectively (Spanish Society of Epidemiology and Family and Community Medicine, 2000). Regarding marital status, 84.6%, 7%, 7.9%, and 0.5% of the mothers were married, divorced, single, and widowed, respectively. In contrast, 91.5%, 4%, and 4.5% of the fathers were married, divorced, and single, respectively. 89.9% of the parents were Spanish, 8.8% were South Americans, 0.7% belonged to other European countries, 0.4% were Asians, and 0.2% were Africans.

**Table 1:** Longitudinal Sample Size

	W1	W2	W3
Youth	692	636	623
Mothers	471	390	-
Fathers	394	321	-

### Measures

#### Youth’s Emotional and Behavioral Difficulties

The Spanish version (Ortuño-Sierra et al., 2015) of the Strengths and Difficulties Questionnaire (Goodman, 1997) was used to assess the emotional and behavioral difficulties reported by the youth. It consisted of 25 items (e.g., “Many worries or often seems worried”; “Many fears, easily scared”; and “Easily distracted, concentration wanders”) answered through a three-point Likert scale (0 = *not true*, 1 = *somewhat true*, 2 = *absolutely true*) and comprised five components: hyperactivity/inattention, emotional symptoms, conduct difficulties, peer relationship difficulties, and prosocial behavior. The mean of hyperactivity/inattention, emotional symptoms, conduct difficulties, and peer relationship difficulties yields one global score, with higher scores reflecting more emotional and behavioral difficulties. Cronbach’s alphas were good for the global score in each wave: *α*_W1_ = 0.77, *α*_W2_ = 0.77, and *α*_W3_ = 0.82.

#### Parental Anxiety Symptoms

Parents answered questions about their anxiety symptoms using the Spanish adaptation (Daza et al., 2002) of the Depression, Anxiety, and Stress Scale (Lovibond & Lovibond, 1995), which was composed of 21 items (e.g., “I was worried about situations in which I might panic and make a fool of myself” and “I felt I was close to panicking”) divided into three subscales: anxiety, depression, and stress symptoms. For this study, only the anxiety subscale was included. Participants responded on a scale ranging from 0 (*did not apply to me at all*) to 3 (*applied to me very much or most of the time*). Cronbach’s alphas were good for maternal (*α*_W1_ = 0.75) and paternal anxiety symptoms (*α*_W1_ = 0.71).

#### Mindful Parenting

Mindful parenting was assessed using the Interpersonal Mindfulness in Parenting Questionnaire (Duncan et al., 2009) reported by parents. This questionnaire comprised 31 items (e.g., “Distracted while engaged with child” and “Busy thinking, not listening to child”) answered on a scale ranging from 1 (*never true*) to 5 (*always true*). The Spanish version has shown adequate psychometric properties (Orue et al., 2023). The Spanish version showed a five-factor structure: non-judgmental acceptance of parenting functioning, compassion of the child, listening with full attention, self-regulation in the parenting relationship, and emotional awareness of the child. For this study, a global score was obtained, with higher scores indicating higher levels of mindful parenting. Cronbach’s alphas were good for both maternal (*α*_W1_ = 0.82, *α*_W2_ = 0.87) and paternal (*α*_W1_ = 0.88, *α*_W2_ = 0.88) mindful parenting.

### Procedure

Once the ethics committee of the University of Deusto approved this study, 11 Biscayan schools were contacted, of which seven agreed to participate in the study. Families provided their informed consent, and 98% allowed their youth to participate. A group of psychologists went to the schools to carry out the data collection in three waves. All invited classes (from fourth to eighth grades) participated in the study. In the classroom, students were informed about the objective of the study and advised that their participation was completely voluntary and confidential and that they could stop participating at any time during the study. They needed approximately 30 min to answer the questions about their emotional and behavioral difficulties. Each student took home two questionnaires to be completed by their parents (one for each parent). Once the parents answered the questions about anxiety symptoms and mindful parenting practices, they returned their questionnaires in a closed envelope to the school. The questionnaires completed by the youth and those completed by their respective parents were linked through a numerical code.

### Data Analysis

Descriptive data and Pearson correlations among the study variables were analyzed using IBM SPSS 27. Little’s missing completely at random test (Little, 1988) was statistically non-significant in both hypothesized models for the mother–youth dyad (χ^2^ = 12.08, *p* = 0.60) and the father–youth dyad (χ^2^ = 7.02, *p* = 0.79). The hypothesized models were tested by path analysis with LISREL 10.2, based on the robust maximum likelihood estimation method with the Satorra–Bentler scaled chi-square (S-B χ^2^) (Jöreskog et al., 2016). The hypothesized models included (a) cross-sectional associations between variables belonging to the same wave; (b) autoregressive paths between youth’s emotional and behavioral difficulties at W1, W2, and W3 and between mindful parenting at W1 and W2; and (c) cross-lagged predictive paths from W1 parental anxiety to W2 mindful parenting, from W1 mindful parenting to W2 youth’s emotional and behavioral difficulties, from W1 youth’s emotional and behavioral problems to W2 mindful parenting and, from W2 mindful parenting to W3 youth’s emotional and behavioral difficulties. Gender was included in the model as a control variable at W1. The significance of the mediational path was tested by bootstrapping (*n* = 5000).

The goodness-of-fit of the models was evaluated using the comparative fit index (CFI), non-normative fit index (NNFI), root mean square error of approximation (RMSEA), and standardized root mean square residual (SRMR). Overall, an acceptable fit is indicated by CFI and NNFI values greater than 0.90 and RMSEA and SRMR values below 0.08 (Little, 2013).

## Results

### Descriptive Data

Table 2 displays the means, standard deviations, and correlations between all study variables. Most of the correlations were in the expected direction and were significant, with some exceptions. No significant correlations were found between W1 youth’s emotional and behavioral difficulties and maternal mindful parenting in W1 and W2; W1 maternal anxiety and W2 youth’s emotional and behavioral difficulties; W2 youth’s emotional and behavioral difficulties and maternal mindful parenting in both W1 and W2; and youth’s emotional and behavioral difficulties with W1 paternal anxiety and mindful parenting. No variable was significantly correlated with age. Gender was significantly associated with youth’s emotional and behavioral difficulties at W1, W2, and W3, indicating that girls scored higher than boys.

**Table 2:** Means, Standard Deviations, and Correlation Coefficients between Study Variables

	1	2	3	4	5	6	7	8	*n*	M	SD
1. Youth’s Emotional and Behavioral Difficulties W1		0.07	−0.03	0.74^***^	−0.14^*^	0.61^***^	0.02	−0.23^***^	240	1.63	0.30
2. Parental anxiety W1	0.12^*^		−0.24^***^	0.02	−0.22^***^	0.02	0.10	−0.08	239	1.18	0.27
3. Mindful parenting W1	−0.09	−0.10		−0.10	0.75^***^	−0.11	0.10	0.03	241	3.73	0.43
4. Youth’s Emotional and Behavioral Difficulties W2	0.70^***^	0.06	−0.07		−0.17^**^	0.74^***^	0.06	−0.19^***^	241	1.57	0.29
5. Mindful parenting W2	−0.04	−0.20^***^	0.67^***^	−0.06		−0.16^**^	0.05	0.07	241	3.71	0.41
6. Youth’s Emotional and Behavioral Difficulties W3	0.60^***^	0.15^**^	−0.13^*^	0.73^***^	−0.12^*^		0.06	−0.20^***^	241	1.57	0.33
7. Youth age	−0.02	−0.02	−0.02	0.08	−0.03	0.05		0.05	241	12.75	1.28
8. Youth gender (0=female; 1=male)	−0.24^***^	−0.06	0.05	−0.23^***^	−0.02	−0.19^***^	0.07		241		
*N*	289	285	290	289	290	289	290	290			
M	1.63	1.25	3.73	1.59	3.72	1.58	12.81				
SD	0.29	0.36	0.41	0.29	0.40	0.32	1.22				

Correlations between variables included in the hypothesized model with mother-youth dyads are below the diagonal. Sample size, means and standard deviations for these variables are included at the bottom. Correlations between variables included in the hypothesized model with mother-youth dyads are above the diagonal. Sample size, means and standard deviations for this section of the diagonal are reported on the right side of the table

*W1* Wave 1, *W2* Wave 2, *W3* Wave 3

^*^p < 0.05. ^**^p < 0.01 ^***^p < 0.001

### Hypothetical Longitudinal Models

Path analysis was conducted to estimate two hypothetical longitudinal models, one including mother–youth dyads and the other including father–youth dyads.

**Fig. 2:**
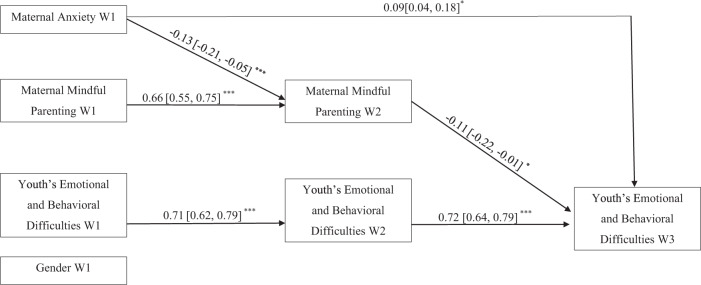
Standardized Coefficients of the Hypothesized Model with Mother-Youth Dyads. W1 = Wave 1; W2 = Wave 2; W3 = Wave 3. The Confidence Intervals are displayed between square brackets. ^*^p < 0.05. ^***^p < 0.001

Path analysis for the hypothetical model estimated with mother–youth dyads revealed some statistically significant paths. Regarding cross-sectional paths between W1 variables, youth’s emotional and behavioral difficulties were positively associated with maternal anxiety. Youth’s emotional and behavioral difficulties and maternal anxiety were negatively associated with maternal mindful parenting and gender. Girls were associated with higher scores on youth’s emotional and behavioral difficulties. W2 maternal mindful parenting and W2 youth’s emotional and behavioral difficulties were significantly but negatively associated. Significant and large autoregressive paths (Cohen, 2013) were obtained for both maternal mindful parenting and youth’s emotional and behavioral difficulties. This result indicates that both maternal mindful parenting and youth’s emotional and behavioral difficulties remain stable over 6 months. In addition, some statistically significant, although small cross-lagged paths, were found. W1 maternal anxiety negatively predicted W2 maternal mindful parenting and W3 youth’s emotional and behavioral difficulties. W2 maternal mindful parenting negatively predicted W3 youth’s emotional and behavioral difficulties. W1 mindful parenting and W1 youth’s emotional and behavioral difficulties did not predict W2 youth’s emotional and behavioral difficulties and W2 mindful parenting, respectively. Gender did not predict any variables at W2 and W3. A more parsimonious model was estimated, including only significant paths. Figure 2 displays the resulting model with standardized coefficients, which obtained excellent fit indexes: χ^2^ (3, *n* = 284) =6.67, *p* = 0.083, RMSEA = 0.06 (95% CI [0.00, 0.13]), CFI = 0.99, NNFI = 0.97, and SRMR = 0.017. To test the significance of the mediating paths, a bootstrapping procedure with 5000 samples was conducted. The results indicate that the mediational path was statistically significant (*b* = 0.014; 95% CI = [0.0004, 0.0323]). W2 maternal mindful parenting mediated the predictive association from W1 maternal anxiety to W3 youth’s emotional and behavioral difficulties.

**Fig. 3:**
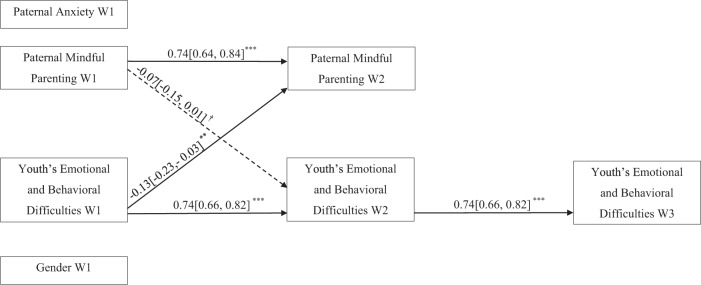
Standardized Coefficients of the Hypothesized Model with Father-Youth Dyads. W1 = Wave 1; W2 = Wave 2; W3 = Wave 3. The Confidence Intervals are displayed between square brackets. ^†^*p* < 0.10. ^**^p < 0.01. ^***^p < 0.001

The path analysis conducted to estimate the hypothetical model with father–youth dyads revealed statistically and positively significant cross-sectional paths between youth’s emotional and behavioral difficulties and paternal anxiety at W1. Associations between maternal mindful parenting and gender with youth’s emotional and behavioral difficulties and paternal anxiety were negative at W1. Girls were associated with higher scores on youth’s emotional and behavioral difficulties. Youth’s emotional and behavioral difficulties were negatively associated with paternal mindful parenting at W2. All the autoregressive paths were statistically significant and showed a large effect size, indicating that paternal mindful parenting and youth’s emotional and behavioral difficulties remained stable over time. In addition, some cross-lagged paths were found. W1 youth’s emotional and behavioral difficulties negatively predicted W2 paternal mindful parenting, whereas paternal mindful parenting marginally and negatively predicted W2 youth’s emotional and behavioral difficulties. The effect size of the cross-lagged paths was small. A more parsimonious model was estimated, which showed excellent fit indexes: χ^2^ (3, *n* = 238) = 4.53, *p* = 0.21, RMSEA = 0.046 (95% CI [0.00, 0.13]), CFI = 1, NNFI = 0.99, SRMR = 0.0014. Figure 3 presents the resulting model with standardized results.

## Discussion

Although the intergenerational transmission of psychological symptoms has been extensively explored, a gap exists in the literature regarding the mechanism of mindful parenting in the transmission of psychological symptoms from parents to youth. To answer one of the central questions about the transmission of psychological symptoms from parents to youth, the mediating role of mindful parenting between parental anxiety and youth’s emotional and behavioral difficulties was evaluated using a longitudinal and multi-informant design. The separated models showed the mediation mechanism of mindful parenting in mother–youth dyads but not in father–youth dyads, suggesting that its role in the transmission of psychological symptoms could be different regarding mothers and fathers.

As hypothesized, maternal anxiety negatively predicted maternal mindful parenting, which in turn predicted higher scores of youth’s emotional and behavioral difficulties. The relationship of the mediation in the hypothesized model with mother–youth dyads and the correlations between the variables used for this model should be highlighted. The correlations between the study variables and maternal anxiety in W1 actually showed a stronger association with emotional and behavioral difficulties in W3 compared with the association with emotional and behavioral difficulties in W1 and W2. These associations could refer to the fact that maternal anxiety does not co-occur with the emotional and behavioral difficulties in youth but rather predicts them at 1 year. Other studies have drawn similar conclusions in this regard, indicating that maternal anxiety is associated not only with the youth’s emotional and behavioral difficulties but also with other developmental outcomes, such as poor socioemotional and adaptive behaviors (Rees et al., 2019; Rogers et al., 2020).

Something similar occurred in the association between maternal mindful parenting and maternal anxiety. Maternal anxiety in W1 was only significantly associated with maternal mindful parenting in W2 and not with maternal mindful parenting in W1. This association reflected that maternal anxiety may precede maternal mindful parenting, as shown in the hypothesized model of mothers. This association was consistent with the conclusion of previous studies indicating that maternal anxiety during pregnancy affects parents’ engagement in mindful parenting (Henrichs et al., 2021). This association in later stages remains scarcely known, such as early adolescence.

Regarding the role of maternal mindful parenting on youth’s emotional and behavioral difficulties, no direct effect was observed from W1 to W2. In the predictive association from W1 maternal anxiety to W3 youth’s emotional and behavioral difficulties, W2 maternal mindful parenting showed a mediating mechanism. In the current study, inconsistent results were found regarding the predictive role of mindful parenting in youth’s emotional and behavioral difficulties. Even though mindful parenting predicted difficulties from W2 to W3, this effect was not found in the path from W1 to W2. This finding could be because mindful parenting affects older youth more and it had an effect when the youth in the current sample were slightly older at W3. Future studies should continue to evaluate this relationship and address these associations to better understand their nature.

In contrast, the hypothesized model with father–youth dyads showed no mediation of mindful parenting in the relationship between paternal anxiety and youth’s emotional and behavioral difficulties. This model resolved some results that may be of great interest, such as the marginal bidirectionality shown between emotional and behavioral difficulties and paternal mindful parenting. This bidirectionality is partial because the path between paternal mindful parenting W1 and emotional and behavioral difficulties W2 was marginally significant. No research has been conducted on the bidirectionality between paternal mindful parenting and youth’s emotional and behavioral difficulties; however, studies have indicated that paternal negative parenting style (e.g., authoritarian style) is highly related to youth’s negative outcomes, such as internalizing and externalizing symptoms (de Maat et al., 2021; Kuppens & Ceulemans, 2019). The effect of youth’s emotional and behavioral difficulties on paternal parenting practices remains largely unknown. Further research is needed in this sense.

The interesting point about this bidirectional result is that the paternal capacity for mindful parenting is affected by the youth’s emotional and behavioral difficulties. Some studies have covered the effects of youth’s emotional and behavioral difficulties on parental outcomes (Sameroff, 2009). A large part of the studies that focused on analyzing parent–youth relationships have evaluated the benefit or risk that parents may pose to their youth’s well-being, even though youth’s emotional and behavioral difficulties may be interfering with the well-being or parenting practices of their parents (Hou et al., 2020).

Notably, despite the fact that no mediation was found in the hypothesized model for fathers in the present study, some important correlations were observed. The correlation table (Table 2) reveals that significant and negative associations appeared between W1 paternal anxiety and W2 paternal mindful parenting and between W2 paternal mindful parenting and W3 youth’s emotional and behavioral difficulties. A significant relationship was found between the variables present in the mediation This relationship was not observed in the hypothesized model.

Differences between the hypothesized models with mother–youth dyads and father–youth dyads were found in the present study, emphasizing the need to evaluate both parents using balanced samples in studies related to parenting and parent–youth relationships. As separate models were conducted for mothers and fathers, the differences between mothers and fathers in the current study cannot be analyzed.

The current study provides some interesting findings concerning youth’s gender. Gender was significantly correlated with a youth’s emotional and behavioral problems, suggesting that girls scored higher in emotional and behavioral problems. The effect size was moderate. In the current study, emotional and behavioral problems were measured with a global score. Future studies that analyze the hypothesized models comparing emotional with behavioral problems are interesting, as previous literature has concluded that boys are likelier to score higher in behavioral problems than girls and that girls score higher than boys in emotional problems (Sanchis-Sanchis et al., 2020).

This study has some limitations. First, the sample selected for each model was small because data from multiple informants were included, which led to many missing cases. Regarding the sample, despite the efforts made to recruit samples that were as balanced as possible in terms of mothers and fathers, an equal sample was not achieved in terms of the number of participating mothers and fathers. This issue could be because mothers tend to participate more frequently in studies related to their offspring, given that they are usually the principal caregivers of their children and are more involved than fathers in youth’s education (Ahmadzadeh et al., 2019). Future research should strive to achieve balanced samples to avoid gender bias in studies related to parenting (Sicouri et al., 2018) and test mediation models with multi-group analysis considering mother–father–youth triads to test whether the model is invariant between mothers and fathers. In addition, actor–partner interdependence models should be included to provide a better conceptualization of the interdependencies in dyadic relationships, such as the interdependence between maternal and paternal mindful parenting (Parent et al., 2021). This would help analyze the differences between mothers and fathers. Future research should assess the study variables at all three time waves to evaluate potential stability and cross-lagged effects. Considering the results of the current study, while maternal mindful parenting explained the intergenerational transmission of psychological symptoms from mothers to youth, paternal mindful parenting was not found to be a mediating mechanism. Future research should cover this gap in the literature (Yaffe, 2020), so it could be determined whether the mediating mechanism of mindful parenting in the intergeneration transmission of psychological symptoms is different when comparing mothers and fathers. This procedure could help to understand whether the development of psychological symptoms among youth differs from previous maternal or paternal psychological symptoms. Future research should evaluate the mediating role of mindful parenting in the relationship between parental anxiety and youth’s emotional and behavioral difficulties in different cultures and groups belonging to different socioeconomic contexts.

## Conclusion

Regarding the theory of the intergenerational transmission of psychological symptoms, there is a research gap in reference to the mediating mechanism of parenting practices for the transmission of psychological symptoms across generations. The current work moves one step forward to one of the central questions regarding this theory, showing that intergenerational transmission could be explained by variables, such as mindful parenting, especially in the case of mothers. This finding provided relevant knowledge to better understand the mechanisms underlying the theory of the intergenerational transmission of psychological symptoms from parents to youth. This study provided a longitudinal and multi-informant design in the study of mindful parenting as a mediating mechanism for the transmission of psychological symptoms from parents to youth, which underlines the importance of analyzing the development of psychological symptoms among youth considering the family context.
